# Hematobiochemical changes in ehrlichiosis in dogs of Anand region, Gujarat

**DOI:** 10.14202/vetworld.2015.713-717

**Published:** 2015-06-06

**Authors:** C. M. Bhadesiya, S. K. Raval

**Affiliations:** Department of Veterinary Medicine, College of Veterinary Science and Animal Husbandry, Anand Agricultural University, Anand, Gujarat, India

**Keywords:** dogs, ehrlichiosis, anti-*Ehrlichia canis* antibodies, hematobiochemical changes

## Abstract

**Aim:**

The present research work was undertaken to study the diagnostic importance of hematobiochemical changes in naturally occurring ehrlichiosis in dogs of Anand region, Gujarat irrespective of their age, breed, and sex.

**Materials and Methods:**

Blood samples from a total of 29 dogs of Anand region of Gujarat state were screened for detection of anti-*Ehrlichia canis* antibodies using Immunocomb^®^ rapid diagnostic kit (Biogal Galed Laboratories, Israel) and subjected to estimation of hematobiochemical parameters by auto hematology analyzers at College of Veterinary Science and Animal Husbandry, Anand. Statistical analysis, interpretation and comparison of hematobiochemical changes with scientific literature was carried out in order to understand the pathophysiology of the disease.

**Results:**

Of 29 dogs, 18 were positive for naturally occurring ehrlichiosis based on the presence of anti-*E. canis* antibodies while 11 were negative. Haematology evinced that the mean values of hemoglobin, total erythrocyte counts, platelet count and packed cell volume in dogs with ehrlichiosis decreased significantly (p<0.01) in comparison to healthy dogs. Among differential leucocyte count, mean values of lymphocytes decreased, neutrophils increased, eosinophils decreased and basophils decreased significantly (p<0.05) in dogs with ehrlichiosis in comparison to healthy dogs while statistically non-significant (p>0.05) difference was observed in values of monocytes in dogs with ehrlichiosis and healthy dogs. Among various red blood cells indices, the mean values of mean corpuscular hemoglobin concentration increased significantly (p<0.01) in dogs with ehrlichiosis in comparison to healthy dogs. Serum biochemistry revealed significant (p<0.01) increase in serum glutamic pyruvic transaminase, serum glutamic oxaloacetic transaminase and creatinine levels as well as decrease in total protein levels in dogs with ehrlichiosis as compared to healthy dogs.

**Conclusion:**

Clinical importance of hematobiochemical changes in 18 natural cases of ehrlichiosis in dogs of Anand region, Gujarat irrespective of their age, breed and sex is discussed, which would aid new insights in diagnosis and therapeutic management.

## Introduction

Ehrlichiosis is one of the major tick-transmitted diseases of dogs and can lead to a wide variety of clinical signs. It is described by the cellular tropism of the infecting organisms, i.e., *Ehrlichia canis* (a small, Gram-negative, coccoid bacterium), which parasitizes cytoplasm of the circulating monocytes in form of distinct clusters termed as “Morulae” [1]. The disease is mainly transmitted by the brown tick of dogs i.e., *Rhipicephalus sanguineus* which passes the organism into blood following bite and is characterized by high fever (104-105°F), anorexia, weakness, lymphadenopathy and epistaxis and edema of dependent parts especially in chronic cases. Once the dog is infected, the course of ehrlichiosis can be divided into three phases, *viz*., acute, subacute and chronic. Subclinical infections of naturally occurring ehrlichiosis in dogs are more common. The chronic stage includes distinct clinical findings with changes in hematological indices including non-regenerative anemia, thrombocytopenia and leucopenia while biochemical abnormalities may represent hypoalbuminemia, hyperglobulinemia and hypergammaglobulinemia [2].

Effective therapeutic management of naturally occurring ehrlichiosis in dogs depends on better understanding of underlying pathophysiology and alterations in organ-functions. Limited investigations have been made in order to evaluate changes in hematological and serum biochemical parameters associated with ehrlichiosis in dog population of Anand region, Gujarat. The present study was carried out in order to evaluate the diagnostic significance of hematobiochemical parameters in naturally occurring ehrlichiosis in dogs of Anand region of Gujarat state irrespective of their age, breed, and sex.

## Materials and Methods

### Ethical approval

Informed consent of the owners was obtained before conducting sample collection procedure from the dogs under study population.

A total of 29 dogs including those presented at the Teaching Veterinary Clinical Complex (TVCC) with clinical signs suggestive of ehrlichiosis and by door to door visits to dog-owners in and around areas of Anand, Gujarat state were clinically examined and 5 ml blood samples from each dog were collected in a sterile anticoagulant vial containing ethylene diamine tetraacetic acid (K_3_EDTA). Blood samples were screened for qualitative detection of anti-*E. canis* antibodies using Immunocomb^®^ Canine Ehrlichia Antibody Test Kit (Biogal Galed Laboratories, Israel), a modified Dot-ELISA to determine canine serum IgG antibody titers to *E. canis* in blood and detection of naturally occurring ehrlichiosis in dog population studied. Incidence was reported with special reference to breed, sex, age and type of housing provided to the dogs. Haematological indices were analyzed by the auto hematology analyzer (BC-2800 Vet, Mindray) at TVCC, College of Veterinary Science and Animal Husbandry, Anand. Furthermore, blood samples were also collected in clot-activator vials to obtain serum samples. Serum biochemical parameters were analyzed by auto-chemistry analyzer using commercial diagnostic kits procured from Crest Biosystem (A Division of Coral Clinical System, Goa) at Department of Veterinary Physiology and Biochemistry, College of Veterinary Science and Animal Husbandry, Anand with standard laboratory protocols, *viz*., estimation of serum glutamic pyruvic transaminase (SGPT) by Reitman and Frankel’s method, serum glutamic oxaloacetic transaminase (SGOT) by modified IFCC method, serum total protein by direct biuret method as well as estimation of serum creatinine by Modified Jaffe’s alkaline picrate method. Hematobiochemical changes in dogs with naturally occurring ehrlichiosis were compared with healthy dogs of Anand region, Gujarat.

### Statistical analysis

The data obtained were subjected to the statistical analysis described by Snedecor and Cochran [3]. The t-test for paired samples having means with unequal variances was carried out. Variables with p<0.05 were considered as statistically “significant,” variables with p<0.01 were considered as statistically “highly significant” and variables with p>0.05 were considered as statistically “non-significant.”

## Results and Discussion

Eighteen out of 29 dogs were positive for naturally occurring ehrlichiosis with high titers of anti-*E. canis* antibodies and the overall incidence of 62.07% while 11 dogs were negative.

### Breed-wise incidence

Breed-wise incidence of naturally occurring ehrlichiosis was recorded higher (22.22%) in German shepherd and Saint Bernard breeds of dogs, each followed by Non-descript breed (16.67%), Doberman pinscher (16.67%), Labrador retriever (11.10%), Pomeranian (05.56%) and Great Dane (05.56%) breeds of dog. German shepherd dog is more predisposed to ehrlichiosis due to inherent breed inability of blast formation and leucocyte migration inhibition factor [4]. To the authors’ knowledge, there are no reports on breed-disposition to the occurrence of ehrlichiosis for other breeds involved in the study.

### Sex and age-wise incidence

Reports suggest that there is no correlation between the incidence of ehrlichiosis and sex as well as the age of dogs [4,5,6]. However, in this study sex-wise incidence of ehrlichiosis was recorded higher (61.11%) in females than males (38.89%) while age-wise incidence was recorded higher (38.89%) among dogs <1 year of age followed by dogs >2 years of age (33.33%) and dogs between 1 and 2 years of age (27.28%).

### Housing pattern-wise incidence

Housing pattern-wise incidence of ehrlichiosis was higher (50.00%) from dogs kept in Pakka house with access to open areas as compared to dogs kept in Kachcha house and field (38.89%) and dogs kept in Pakka houses (11.11%). To the authors’ knowledge, there are no reports correlating the incidence of ehrlichiosis in dogs with special reference to types of housing provided to the dogs.

### Clinical signs

The most significant clinical findings associated with naturally occurring ehrlichiosis were hyperthermia in 18 (100.00%), tick infestation in 18 (100.00%), lymphadenopathy in 17 (94.44%), coat abnormalities in 11 (61.11%), anorexia in 11 (61.11%), pallor of mucosae in 10 (55.56%), weight loss in 10 (55.56%), petechial hemorrhages over abdomen in 08 (44.47%) and epistaxis in 02 (11.11%) dogs.

The study was aimed to evaluate hematobiochemical indicators in dogs positive for naturally occurring ehrlichiosis irrespective of their age, breed and sex in comparison with normal healthy dogs as shown in Table-1.

**Table-1:** Case distribution and hematobiochemical alterations (mean±SE) associated with ehrlichiosis in dogs.

Case distribution (n=18)		

Criteria	Number of dogs	Percentage
Breed-wise incidence of ehrlichiosis		
Non-descript breed	03	16.67
German Shepherd dog	04	22.22
Labrador retriever	02	11.10
Saint Bernard dog	04	22.22
Doberman pinscher	03	16.67
Pomeranian	01	05.56
Great Dane	01	05.56
Sex-wise incidence of ehrlichiosis		
Male	07	38.89
Female	11	61.11
Age-wise incidence of ehrlichiosis		
<1 year of age	07	38.89
1-2 years of age	05	27.28
>2 years of age	06	33.33
Housing pattern-wise incidence of ehrlichiosis		
Kachcha house and field	07	38.89
Pakka house	02	11.11
Pakka house with access to open areas	09	50.00

**Hematobiochemical parameters (mean±SE) in dogs with ehrlichiosis**		

**Parameter**	**Healthy dogs (n=11)**	**Dogs with Ehrlichiosis (n=18)**

Hematological parameters		
Hb (g/dl)	13.61±00.73	09.94±00.53**
TEC (×10^6^/µl)	07.83±00.41	05.20±00.24**
TLC/WBCs (×10^3^/µl)	10.73±00.24	09.81±01.64
Lymphocytes (%)	32.49±01.45	21.79±02.60*
Neutrophils (%)	58.15±03.14	70.67±02.73*
Eosinophils (%)	03.91±00.49	01.78±00.45*
Basophils (%)	00.80±00.16	00.58±00.10
Monocytes (%)	02.91±00.51	04.68±00.41*
Platelet count (×10^5^/µl)	316.73±23.48	124.72±12.93**
PCV (%)	45.01±02.10	28.70±01.38**
MCV (fl)	65.51±00.73	65.00±00.66
MCHC (g/dl)	33.70±00.55	38.09±00.76**
MCH (pg)	22.77±00.46	23.25±00.52
Serum biochemical parameters		
Total protein (g/dl)	05.82±00.17	04.01±00.13**
SGPT (IU/L)	50.66±05.01	87.87±01.97**
SGOT (IU/L)	51.28±02.71	65.02±00.60**
Creatinine (mg/dl)	01.30±00.03	01.80±00.02**

* (p<0.05)

** (p<0.01)

SGOT=Serum glutamic oxaloacetic transaminase, SGPT=Serum glutamic pyruvic transaminase, MCHC=Mean corpuscular hemoglobin concentration, MCH=Mean corpuscular hemoglobin, MCV=Mean corpuscular volume, PCV=Packed cell volume, WBCs=White blood cells, TLC=Thin layer chromatography, TEC=Total erythrocyte count, SE=Standard error, Hb=Hemoglobin

### Haematological parameters

Levels of hemoglobin (Hb), total erythrocyte count (TEC), platelet count and packed cell volume (PCV) decreased significantly (p<0.01) in dogs with ehrlichiosis than healthy dogs. Decrease in Hb levels were in agreement with reports of Castro *et al*. [7], Sharma *et al*. [8], Dixit *et al*. [9], Srikala *et al*. [10] and Bhardwaj [11]. Decreased TEC levels were in agreement with reports of Castro *et al*. [7], Sharma *et al*. [8], Bhardwaj [11] and Oliveira [12]. Decreased Hb and TEC levels could be due to epistaxis, petechial hemorrhages, myelosuppression or due to severe anemia [11]. Decreased platelet counts (i.e. thrombocytopenia) in ehrlichiosis were in agreement with reports of Srikala *et al*. [10], Bhardwaj [11], Oliveira [12], Waner *et al*. [13], Niwetpathomwat *et al*. [14], Irwin [15], Nakaghi *et al*. [16], Srivastava and Srivastava [17] and Agnihotri *et al*. [18]. Decreased levels of Hb, TEC, and platelet counts were suggestive of underlying blood coagulopathy in dogs with naturally occurring ehrlichiosis.

The difference between total leucocyte count in dogs with ehrlichiosis and healthy dogs was statistically non-significant. However, Srikala *et al*. [10], Bhardwaj [11], Oliveira [12], Waner *et al*. [13], Niwetpathomwat *et al*. [14], Irwin [15], Nakaghi *et al*. [16], Srivastava and Srivastava [17] and Shipov *et al*. [19] reported presence of significant leucopenia in dogs with ehrlichiosis. Among differential leucocyte count, levels of lymphocytes decreased significantly (p<0.05) in dogs with ehrlichiosis than healthy dogs. These findings were in agreement with lymphocytopenia associated with ehrlichiosis in dogs as reported by Castro *et al*. [7] and Oliveira [12]. However, Dixit *et al*. [9] reported a significant increase in lymphocytes in dogs with ehrlichiosis. Lymphocytopenia is suggestive of myelosuppression in ehrlichiosis [2]. Levels of neutrophils increased significantly (p<0.05) in dogs with ehrlichiosis than healthy dogs. These findings were in agreement with Castro *et al*. [7]. However, Dixit *et al*. [9] reported a significant decrease in neutrophils (%) in dogs with ehrlichiosis. Neutrophilia in ehrlichiosis may represent underlying co-infection [18]. Levels of eosinophils decreased significantly (p<0.05) in dogs with ehrlichiosis than healthy dogs. These findings were in agreement with Castro *et al*. [7] and Srikala *et al*. [10]. The difference between basophils counts in dogs with ehrlichiosis and healthy dogs was statistically non-significant. Levels of monocytes increased significantly (p<0.05) in dogs with ehrlichiosis than healthy dogs. These findings were in agreement with Castro *et al*. [7] while Oliveira [12] recorded significant decrease in levels of monocytes in cases positive for ehrlichiosis.

Among red blood cells indices, values of mean corpuscular Hb concentration (MCHC) increased significantly (p<0.01) in dogs with ehrlichiosis than healthy dogs. These findings were in agreement with Castro *et al* [7]. The difference between values of mean corpuscular volume in dogs with ehrlichiosis and healthy dogs was statistically non-significant. The difference between values of mean corpuscular Hb (MCH) in ehrlichiosis positive dogs and healthy dogs was statistically non-significant. However, Mulla [20] recorded significant (p<0.05) decrease in levels of MCH in dogs with ehrlichiosis.

### Serum biochemical parameters

Among various serological parameters, the levels of total protein decreased significantly (p<0.01) in dogs with ehrlichiosis than healthy dogs. Results suggests hypoproteinemia associated with ehrlichiosis in dogs, which is in agreement with Castro *et al*. [7], Srikala *et al*. [10], Srivastava and Srivastava [17], Agnihotri *et al*. [18] and Harrus *et al*. [21] while Bhardwaj [11], Irwin [15] and Weiser *et al*. [22] recorded significant increase in total protein levels in dogs with ehrlichiosis. Levels of SGPT increased significantly (p<0.01) in dogs with ehrlichiosis than healthy dogs. These findings were in agreement with Srikala *et al*. [10], Bhardwaj [11] and Agnihotri *et al*. [18], Katyal [23] and Mallapur [24]. Levels of SGOT increased significantly (p<0.01) in dogs with ehrlichiosis than healthy dogs. These findings were in agreement with Bhardwaj [11] and Agnihotri *et al*. [18]. Increased levels of SGPT and SGOT are indicative of hepatic dysfunction leading to hypoproteinemia in dogs with ehrlichiosis [14,18]. Levels of creatinine increased significantly (p<0.01) in dogs with ehrlichiosis as compared to healthy dogs. These findings were in agreement with Srivastava and Srivastava [17], Agnihotri *et al*. [18], Katyal [23] and Mallapur [24] while Patil [25] recorded significant decrease in levels of creatinine. The increase in creatinine levels may be due to immune complex-mediated glomerulonephritis indicating renal involvement in dogs with ehrlichiosis [18].

## Conclusion

The overall incidence of ehrlichiosis, based on Dot-ELISA rapid diagnostic kit, was recorded as 62.07%. The highest breed-wise, sex-wise, age-wise and housing pattern-wise incidence of ehrlichiosis was recorded in German shepherd breed of dog (22.22%), females (61.11%), young dogs with age <1 year (38.89%) and dogs kept in pakka house with access to open areas (50.00%), respectively. Hematobiochemical changes in 18 dogs positive for naturally occurring ehrlichiosis based on the Dot-ELISA based rapid diagnostic kit irrespective of their age, breed and sex are discussed which evinced lowered platelet counts, Hb levels, TEC, PCV, eosinophils and lymphocytes, as well as increased neutrophils, monocytes and MCHC as compared to healthy dogs. Dogs with ehrlichiosis showed increased levels of SGPT, SGOT and serum creatinine suggestive of hepatic and renal involvement in the pathophysiology of the disease. Results suggest that estimation of hematobiochemical parameters holds equal importance in a complete diagnostic approach for dogs with naturally occurring ehrlichiosis and should be considered prior to the therapeutic approach.

## Authors’ Contributions

CMB: Conducted the research work, which includes experimental design, a collection of blood and serum samples, use of the rapid diagnostic test, estimation of hematobiochemical parameters and statistical analysis, preparing and drafting the manuscript. SKR: Provided guidance during the entire experiment and corrected manuscript. Both authors read and approved the final manuscript.

## References

[1] Ristic M, Holl C.J, Woldehiwet Z, Ristic M (1993). Canine ehrlichiosis. Rickettsial and Chlamydial Diseases of Domestic Animals.

[2] Ettinger S.J, Feldman E.C (2005). Diseases of the dog and cat. Textbook of Veterinary Internal Medicine.

[3] Snedecor G.W, Cochran W.G (1994). Statistical Methods.

[4] Huxsoll D.L, Amyx H.L, Hemelt I.E, Hildebrandt P.K, Nims R.M, Gochenour W.S (1972). Laboratory studies of tropical canine pancytopenia. Exp. Parasitol.

[5] Tresamol P.V, Dinakaran M, Suresh S (1998). Serological diagnosis of *Ehrlichia canis* infection in dogs. Cherion.

[6] Rahman W.A, Ning C.H, Chandrawathani P (2010). Prevalence of canine ehrlichiosis in Perak State, Penisular Malaysia. Top. Biomed.

[7] Castro D.B, Machado R.Z, Tomaz de Aquino L.P, Alessi A.C, Costa M.T (2004). Experimental acute canine monocytic ehrlichiosis:Clinicopathological and immunopathological findings. Vet. Parasitol.

[8] Sharma D.K, Bhuyan D, Phangchoo C.V, Baishya B.C (2010). Efficacy of oxytetracycline and doxycycline in the treatment of canine ehrlichiosis. Intas Polivet.

[9] Dixit A.K, Dixit P, Sukla P.C (2012). Canine monocytic ehrlichiosis and its therapeutic management in a dog. Intas Polivet.

[10] Srikala D, Satish Kumar K, Amruth Kumar V.V, Tirumala Rao D.S (2012). Clinical and therapeutic aspects of canine monocytic ehrlichiosis. Indian J. Vet. Med.

[11] Bhardwaj R.K (2013). Therapeutic management of acute canine monocytic ehrlichiosis. Indian Vet. J.

[12] Oliveira D (2000). *Ehrlichia canis* antibodies detection by “Dot-ELISA” in naturally infected dogs. Rev. Braz. Parasitol. Vet.

[13] Waner T, Harrus S, Jongejan F, Bark H, Keysary A, Cornelissen A.W (2001). Significance of serological testing for ehrlichial diseases in dogs with special emphasis of canine monocytic ehrlichiosis caused by *Ehrlichia canis*. Vet. Parasitol.

[14] Niwetpathomwat A, Techangamsuwan S, Suvamavibhaja S (2005). A retrospective study of the clinical hematology and biochemistry of canine ehrlichiosis in an animal hospital population in Bangkok, Thailand. J. Clin. Pathol.

[15] Irwin P.J (2007). Pups, PCRs and Platelets *Ehrlichia* and Anaplasma Infections of dogs in Australia and Overseas.

[16] Nakaghi A.C, Machado R.Z, Costa M.T, Andre M.R, Baldani C.D (2008). Canine ehrlichiosis:Clinical, haematological, serological and molecular aspects. Cien. Rural Santa Maria.

[17] Srivastava M.K, Srivastava A (2011). Canine ehrlichiosis in dog. Indian J. Vet. Med.

[18] Agnihotri D, Khurana R, Jain V.K, Singh G (2012). Concurrent infection of *Ehrlichia canis* and ancylostomosis in a dog. Indian Vet. J.

[19] Shipov A, Klemet E, Reuveni-Tager L, Waner T, Harrus S (2008). Prognostic indicators of canine monocytic ehrlichiosis. Vet. Parasitol.

[20] Mulla F (2007). Scintigraphic study of doxycycline nanoparticulate in rabbits and assessing its efficacy in canine ehrlichiosis.

[21] Harrus S, Kenny M, Miara L, Aizenberg I, Waner T, Shaw S (2004). Comparison of simultaneous splenic sample PCR with blood sample PCR for diagnosis and treatment of experimental *Ehrlichia canis* infection. Antimicrob. Agents Chemother.

[22] Weiser M.G, Thrail M.A, Dulton R, Beck E.R, Wise L.A, Vansteenhouse J.L (1991). Granular lymphocytosis and hyperproteinemia in dogs with chronic ehrlichiosis. J. Am. Anim. Hosp. Assoc.

[23] Katyal D.P (2000). Studies on the prevalence of canine blood parasitic infections with special reference to the diagnosis and chemotherapy of *Ehrlichia canis*.

[24] Mallapur S.S (2002). Studies on ehrlichiosis in dogs in Mumbai.

[25] Patil S.L (2009). Efficacy of targeted nanoparticulate doxycycline in canine ehrlichiosis.

